# Cognitive-constructivist Approach in Medical Settings: The Use of Personal Meaning Questionnaire for Neurological Patients’ Personality Investigation

**DOI:** 10.3389/fpsyg.2017.00582

**Published:** 2017-04-11

**Authors:** Barbara Poletti, Laura Carelli, Annalisa Lafronza, Federica Solca, Andrea Faini, Andrea Ciammola, Monica Grobberio, Vanessa Raimondi, Rita Pezzati, Rita B. Ardito, Vincenzo Silani

**Affiliations:** ^1^Department of Neurology and Laboratory of Neuroscience, IRCCS Istituto Auxologico ItalianoMilan, Italy; ^2^Department of Cardiovascular, Neural and Metabolic Sciences, IRCCS Istituto Auxologico ItalianoMilan, Italy; ^3^Laboratory of Clinical Neuropsychology, Department of Neurology – ASST LarianaComo, Italy; ^4^Department of Neurology and Clinical Psychology Services, ASST CremaCrema, Italy; ^5^University of Applied Sciences and Arts of Southern SwitzerlandManno, Switzerland; ^6^Centro Terapia CognitivaComo, Italy; ^7^Center for Cognitive Science, Department of Psychology, University of TurinTurin, Italy; ^8^Department of Pathophysiology and Transplantation and “Dino Ferrari” Center, University of Milan Medical SchoolMilan, Italy

**Keywords:** Amyotrophic Lateral Sclerosis, cognitive-constructivist psychotherapy, multiple sclerosis, personality, personal meaning questionnaire, primary headache

## Abstract

**Objective:** The cognitive-constructivist psychotherapy approach considers the self as a continuous regulation process between present and past experience, in which attributions of meaning is characterized by the use of internal rules. In this conception, everyone would be driven by a specific inner coherence called Personal Meaning Organization (PMO). Such approach has never been applied to neurological patients by means of *ad hoc* developed tools. We performed an explorative study aimed to characterize personality styles in different neurological conditions within the theoretical framework of cognitive-constructivist model.

**Materials and Methods:** Three groups of neurological patients (Amyotrophic Lateral Sclerosis, Multiple Sclerosis, Primary Headache) and a sample of healthy participants, each composed by 15 participants, for a total of 60 participants, were recruited. The Personal Meaning Questionnaire (PMQ), an Italian questionnaire assessing PMOs construct, and other clinical tools for psychological and quality of life assessment were administered to all subjects.

**Results:** The main finding concerned the detection, across all clinical conditions, of a higher prevalence of phobic personality style, with Amyotrophic Lateral Sclerosis showing a relevant prevalence of such PMO with respect to all other neurological conditions and controls. However, with respect to controls, in all clinical conditions, PMQ highlighted a tendency, even if not statistically significant, to codify experience by means of specific cognitive and emotional patterns.

**Conclusion:** Our findings represent the first contribution towards understanding the personality profiles of patients affected by neurological conditions according to cognitive-constructivist theory.

## Introduction

Within the theoretical background of cognitive-constructivism, the introduction of concepts such as ‘cognitive organization’ and ‘personal meaning organization’ (PMO) provided a general and explanatory theory of personality (Guidano, 1981, 1987; Guidano and Liotti, 1983). Psychotherapy based on cognitive-constructivist approach considers the self as a continuous regulation process between past and present experience, where the attribution of meaning is based on individual rules aimed to maintain internal consistency, in a dynamic dialectic between immediate experience (implicit) and the reorganization of such experience (explicit), as well as to manage perturbations arising from the environment. According to such theory, everyone is oriented by an internal consistency provided by ‘personal meaning organization styles’. Four PMOs have been described: depressive, phobic, eating disorder and obsessive (Guidano, 1981, 1987). In the latest elaborations of this theory, such organizations are represented as ‘personality styles’ which can be observed also in non-clinical populations (Arciero, 2002; Picardi et al., 2003).

The Eating Disorder Organization (ED) identifies individuals who tend to select internal states and opinions based on an external point of reference; they are thus characterized by a strong attention to expectations perceived in others, a need for approval, a sensitivity to judgment and to criticism.

The Obsessive Organization (OBS) identify individuals where the sense of self is based primarily upon conscious control of behavior and thinking, both of which are expected to match abstract principles; they present themes of responsibility, anticipatory control, equity, order, certainty and coherence.

The Phobic Organization (PHOB) is characterized by a vulnerable sense of self that interprets emotional states as impediments to action. Such individuals are characterized by themes of freedom and invincibility commonly entangled with those of health, friendship, affective stability.

The Depressive Organization (DEP) is characterized by a sense of personal ineptitude on the affective level and a constant experience of solitude within one’s primary attachment. A tendency to perceive a continuous and latent sense of loss in life events can be observed in such individuals.

The development by Picardi and Mannino (2001), Picardi (2003), and Picardi et al. (2003) of the Personal Meaning Questionnaire (PMQ), aimed at assessing the construct of PMO, provided new methods of investigation within cognitive-constructivist paradigm. A successive experimental study (Picardi et al., 2004) further supported the convergent validity of such tool against other widely used personality questionnaires, such as the Temperament and Character Inventory (TCI-125; Cloninger et al., 1994) and the Big Five Questionnaire (BFQ; Caprara et al., 1993). Moreover, it has also been employed in order to highlight associations between different personality styles and cognitive evaluation of emotional stimuli in healthy subjects, by means of functional magnetic resonance imaging - fMRI (Bertolino et al., 2005; Rubino et al., 2007). However, such instrument has never been administered to patients affected by neurological diseases.

In the past, several authors investigated personality styles in neurological disease in order to identify the possible influence of permanent emotional and psychological aspects on disease onset, course and management (Aben et al., 2002; Bragazzi, 2013). However, less attention has been paid to the identification of specific personality profiles characterizing patients with neurological diseases. Within this field, studies focused on patients with chronic and disabling disease, such as Parkinson’s disease and Multiple Sclerosis (MS), as well as patients suffering from chronic pain, such as headache and migraine. Fewer findings, even if promising, concern another disabling neurological condition, that is Amyotrophic Lateral Sclerosis (ALS), where a complex interplay between cognitive, emotional and neurobiological aspects has been recently highlighted (Goldstein and Abrahams, 2013).

Amyotrophic Lateral Sclerosis can be defined as a neurodegenerative disorder characterized by progressive muscular paralysis reflecting degeneration of motor neurons (MNs) in the primary motor cortex, brainstem, and spinal cord (Silani et al., 2011). Cognitive impairment occurs in approximately 30% of ALS patients with about 10% of patients presenting frontotemporal dementia, mostly the behavioral variant (Beeldman et al., 2015).

Clinical manifestations of MS are much more variable, both concerning onset features and progression. MS is a disease of the central nervous system, which affects the brain, spinal cord, and the optic nerves. Four different MS subtypes have been identified and defined: relapsing-remitting (RRMS), secondary-progressive (SPMS), primary-progressive (PPMS), and progressive-relapsing (PRMS) (Lublin and Reingold, 1996). Afterward the clinical isolated syndrome (CIS), MS with benign course and MS with malign course forms have been recognized (Trojano and Paolicelli, 2001; Polman et al., 2011; Lublin et al., 2014).

The Primary Headache (PH) syndrome, frequently observed in clinical practice, could manifest as migraine, tension headache or cluster headaches, with episodic or chronic course (Headache Classification Committee of the International Headache Society, 1988, 2004).

The association between MS, headache and ALS and specific psychological disorders has been discussed for a long time, but only in the last decades systematic studies have been performed in order to better define these aspects. In MS, psychological problems are often described as a specific consequence of adaptation to physical and sensory impairments related to the chronic and progressively disabling disease (Baretz and Stephenson, 1981; Johansson et al., 2016). Some authors, however, reported that such emotional aspects cannot be closely correlated with the diagnosis (Withlock and Siskind, 1980), highlighting the presence of independent characteristics contributing to them. The most frequently described psychological disorder in MS is depression, with a prevalence rate of 50% reported after diagnosis (Sadovnick et al., 1991; Feinstein, 2011). The incidence of this disorder in MS is significantly increased both compared to other chronic diseases (Minden et al., 1987; Patten et al., 2003; Even et al., 2004) and to other neurological conditions (Rabins et al., 1986). A biologic hypothesis suggests that onset of depression in MS may be induced by temporal regions lesions secondary to the disease (Mohr et al., 2003). Besides, also specific anxiety disorders have been described in MS patients (Korostil and Feinstein, 2007). Similarly to MS, also headache appears to be closely correlated with depressive symptoms, particularly when disease becomes chronic causing feelings of inadequacy, negative thinking, diminished interest for environment and relationships, affecting patients’ quality of life – QoL (Blomkvist et al., 2002; Manack et al., 2011). In the last decade, however, it has been suggested that depressive and anxious features, as well as other psychological aspects, may be a distinctive characteristic of this population of patients, instead of a consequence of experimenting chronic pain (Mongini et al., 2000; Torelli et al., 2008; Berry and Drummond, 2014; Bottiroli et al., 2016; Muñoz et al., 2016). In ALS population, by contrast, Major Depressive Disorder (MDD) seems to be an uncommon result, with a prevalence similar to that of the adult general population (Norris et al., 2010). Of greater importance appears to be the tendency of ALS patients to actively negate the disease, so relegating it out of conscious awareness (Brown and Mueller, 1970; Grossman et al., 2006). In ALS, the so-called ‘*disability paradox’* concerns evidence that subjective QoL and emotional well-being in ALS patients are comparatively good and partially unrelated to physical functioning and disease severity and progression (Kurt et al., 2007; Lulé et al., 2008, 2012; Norris et al., 2010).

The possible existence of a specific personality profile for these three diseases has been suggested. In particular, personality aspects have been depicted in MS, according to several studies (Invernizzi et al., 1988; Merkelbach et al., 2003; Fishman et al., 2004; Incerti et al., 2015; Schreiber et al., 2015; Mohamadi et al., 2016; Strober, 2016; Zarbo et al., 2016), even if some of them focused only on euphoric symptoms and emotional lability (Feinstein, 2004). Moreover, recent findings suggest a possible role of personality even in clinical neuroscience of MS (Benedict et al., 2013).

Patients with migraine have been described as having particular difficulties in recognition of their own emotions, showing high levels of anxiety and frequent concerns about their health (Yucel et al., 2002; Norton and Asmundson, 2004), suggesting such features as distinctive patients’ characteristics (Torelli et al., 2008; Berry and Drummond, 2014).

Concerning ALS, even if some studies suggested the presence of a specific personality style characterizing such patients (Brown and Mueller, 1970; Grossman et al., 2006), some others were unable to support such findings (Houpt et al., 1977; Peters et al., 1978). Such conflicting literature still remains a unresolved issue.

To assess personality profiles, different tools have been employed in the past in MS, ALS, and PH diseases. Between them, it is worthwhile mentioning: Temperament and Character Inventory (TCI, Abbate-Daga et al., 2007; Fazekas et al., 2013; Verdolini et al., 2015), Neo Personality Inventory (NEO-PI; Krampe et al., 2008; Berry and Drummond, 2014; Incerti et al., 2015), Millon Clinical Multiaxial Inventory III (MCMI-III; Mohamadi et al., 2016), Eyesenk Personality Questionnaire (EPQ; Aaseth et al., 2011), and the Iowa Scale of Personality Change (ISPC; Waldron et al., 2014).

Moreover, a widely used personality inventory, the Minnesota Multiphasic Personality Inventory (MMPI; Hathaway and McKinley, 1997) has also been employed in such neurological populations highlighting a specific bias in ALS and MS patients, where personality profile appears to be influenced by an overestimation of the ‘psychosomatic’ profile due to the specific content of some items. Accordingly, some authors had discussed the opportunity to use an *ad hoc* statistical correction for neurological patients in order to balance disease related symptoms influence on personality description (Nelson et al., 2003; Incerti et al., 2016).

Overall, despite different tools have been used, no conclusive data were obtained concerning the role of personality in the above mentioned neurological diseases, especially when considering the existence of a disease specific personality profile.

The use of PMQ, and the underlying cognitive-constructivist model, aims to take into account the complexity of human experience and not only specific personality dimensions; it demonstrated to be independent from changes across time in levels of depression and anxiety and to have a strong convergent validity with other traditional personality inventories (Picardi et al., 2004). For these reasons, it can be considered as a useful and comprehensive measure to be employed with neurological patients. By means of PMQ, the present study aims to explore personality features of neurological patients with ALS, MS, or PH in order to possibly identify specific personality profiles characterizing such conditions within the cognitive-constructivist frame. The study of these three clinical populations could provide suggestions on factors influencing chronic or degenerative neurological disorder subjective experience. According to psychological and quality of life components involved in chronic neurological diseases, also such dimensions together with personality were explored. Such investigation could possibly deeply characterize clinical samples, thus better defining disease-specific psychological features from a comprehensive perspective.

## Materials and Methods

### Subjects

Three different groups of patients were recruited: 15 patients with ALS, 11 with spinal onset, and 4 with bulbar onset (5 females; mean age: 60.0 + 10.3 years old; mean education 9.0 + 3.6 years); 15 patients with RRMS (11 females; mean age 41.3 + 7.0 years old; mean education 12.3 + 3.2 years); 15 patients with PH, of which 5 with tension-type headache, 2 with migraine, and 8 with mixed headache (10 females; mean age 36.1 + 15.0 years old; mean education 12.7 + 4.0 years old). Diagnosis of possible or probable ALS and MS was made according to El Escorial (Brooks et al., 2000) and McDonald (McDonald et al., 2001) criteria, respectively. Patients suffering from PH were diagnosed according to ICDH criteria (Headache Classification Committee of the International Headache Society, 1988).

Control group was composed by 15 healthy participants (9 females, mean age 42.3 + 12.0 years old; mean education 12.5 + 2.3 years old), not affected by any chronic clinical condition or suffering from any psychiatric or neurological disorder.

The study protocol was approved by the Ethics Committee of IRCCS Istituto Auxologico Italiano and all eligible subjects received verbal and written information about the study. All participants signed an informed consent, according to the Declaration of Helsinki.

### Psychological and Personality Assessment

Each subject underwent a battery of measures investigating psychological, QoL and personality features: The Personal Meaning Questionnaire (PMQ), the State-Trait Anxiety Inventory – Form Y (STAI-Y), the Beck Depression Inventory (BDI), the Symptom Check List 90 (SCL-90), and the Short-Form Health Survey 36 (SF-36).

PMQ consists of a self-report questionnaire, developed by Picardi and Mannino (2001), Picardi (2003), and Picardi et al. (2003), aimed at empirically support the validity of the ‘personal meaning organizations’ theory developed by Guidano (Guidano, 1987). Such tool intends to explore thoughts, feelings and behaviors as expected in the different PMOs. It is composed by 68 items, to be answered according to the level of agreement/disagreement or to a 5-points Likert scale.

The State-Trait Anxiety Inventory-Y (STAI-Y) (Spielberger et al., 1989) is aimed at detecting and evaluating anxiety symptoms, concerning both state (STAI-Y1) and trait (STAI-Y2) anxiety components. It is made of 40 questions, to be answered according to both intensity and frequency of symptoms.

The BDI (Beck et al., 1979) evaluates depressive symptomatology, concerning somatic, emotional, cognitive and motivational components. It is composed by 21 items, each of them corresponding to a symptoms cluster useful to identify severity of depression.

SCL-90 inventory (Derogatis et al., 1973) was employed in order to evaluate a broad range of psychopathological symptoms. Such scale contains 90 items, highlighting mental disease according to 9 symptoms dimensions that correspond to: somatization (SOM), obsessive–compulsive (O–C), interpersonal sensitivity (IS), depression (DEP), anxiety (ANX), hostility (HOS), phobic anxiety (PHOB), paranoid ideation (PAR), and psychoticism (PSY). A further questionnaire, the SF-36 (Apolone et al., 1997), was also administered in order to detect health related QoL, consisting of a multidimensional tool made of 36 questions. Items concern 8 different health domains: physical functioning (PF), role limitations due to physical health (RP), bodily pain (BP), general health (GH), energy (EN), social functioning (SF), role limitations due to emotional problems (RE), mental health (MH). The SF-36 has eight scaled scores; the scores are weighted sums of the questions in each section. Scores range from 0 to 100, with lower scores suggesting more disability.

### Statistical Analysis

Descriptive data are reported as means ± standard deviations for continuous variable and as absolute numbers for categorical variable. Distribution of the variables in terms of proximity to normal was detected by Shapiro-Wilk test; the homogeneity of variances was performed with Bartlett test. For the variables normally distributed and homoscedastic, the ANOVA with pairwise *t*-test as *post hoc* was used; otherwise a Kruskal–Wallis rank sum test was employed with pairwise Wilcoxon rank sum test as *post hoc*. For multiple comparisons, the algorithm which controls the expected rate of false-positive results for all positive results (false discovery rate) was used. For each individual the QSP profile was calculated using the maximum values among the following variables: ED, DEP, PHOB, and OBS. The QSP prevalence was tested using the Fisher exact test. Finally, a Spearman’s rank correlation analysis (*r*_s_) was employed to examine relationships between scores obtained at the different questionnaires. An α level of 0.05 was used for all hypothesis tests. All data analyses were performed using R Core Team (2015), Vienna, Austria.

## Results

### Characteristics of Subjects

See **Table 1** for descriptive statistics concerning psychological, QoL, and personality features of neurological patients and controls.

**Table 1:** Mean (standard deviation, SD) scores obtained by neurological patients and control subjects at psychological questionnaires.

	MS	PH	ALS	CONTROL	*p*-value
STAI-Y1	42.67 (14.95)*	46.60 (13.94)**	46.87 (13.22)**	30.93 (6.62)	0.003
STAI-Y2	46.73 (12.87)**	44.53 (12.01)**	41.40 (10.91)*	30.87 (6.99)	0.001
BDI	12.47 (10.70)**	14.40 (13.78)**	16.20 (9.37)***	2.67 (2.35)	<0.001
SF-36 PF	72.00 (23.89)	84.67 (18.27)	37.00 (29.20)***	90.33 (10.60)	<0.001
SF-36 RP	41.67 (41.90)***	40.00 (38.73)***	35.00 (40.97)***	98.33 (6.45)	<0.001
SF-36 BP	58.73 (28.07)*	36.07 (23.19)***	61.40 (26.79)*	83.53 (19.84)	<0.001
SF-36 GH	42.67 (24.63)***	56.20 (14.84)*	38.40 (24.31)***	76.67 (11.78)	<0.001
SF-36 EN	50.67 (22.82)	43.00 (23.05)	52.33 (24.92)	64.33 (15.80)	0.075
SF-36 SF	68.53 (30.13)	51.40 (20.95)***	61.93 (25.62)**	88.53 (13.61)	0.001
SF-36 RE	53.40 (43.32)**	42.27 (44.53)**	48.87 (43.45)**	93.40 (13.67)	0.004
SF-36 MH	58.53 (19.46)**	52.00 (25.12)**	59.20 (22.94)*	79.73 (12.78)	0.002
SCL-90 SOM	14.67 (9.36)**	17.53 (10.79)**	12.00 (9.20)**	4.87 (4.31)	0.002
SCL-90 O-C	11.40 (9.26)*	12.73 (8.84)*	6.87 (4.49)*	3.33 (3.54)	0.006
SCL-90 IS	7.07 (4.53)	8.20 (8.40)	5.60 (5.21)	2.93 (3.83)	0.043
SCL-90 DEP	14.73 (11.73)**	14.40 (11.93)**	12.47 (11.20)***	2.93 (2.81)	<0.001
SCL-90 ANX	9.20 (7.02)**	10.20 (9.00)**	6.60 (6.66)*	2.00 (2.59)	0.002
SCL-90 HOS	4.80 (3.75)**	4.93 (5.04)**	2.67 (1.95)*	0.87 (0.99)	< 0.001
SCL-90 PHOB	1.73 (2.15)*	2.87 (3.38)*	3.53 (5.329***	0.27 (0.59)	0.002
SCL-90 PAR	4.40 (2.59)	6.60 (6.44)	3.27 (2.28)	2.20 (2.93)	0.056
SCL-90 PSY	5.87 (6.15)**	7.33 (7.88)**	4.00 (2.42)**	1.33 (2.23)	0.004
PMQ ED	51.40 (11.67)*	53.93 (12.70)*	49.47 (11.79)	40.80 (11.89)	0.022
PMQ DEP	43.13 (10.67)*	44.07 (14.32)*	40.60 (8.25)	32.80 (9.06)	0.025
PMQ PHOB	58.33 (8.37)	64.00 (9.07)	65.00 (7.46)*	56.87 (7.56)	0.017
PMQ OBS	55.07 (8.75)	59.60 (9.77)	56.53 (7.30)	53.87 (8.15)	0.297

^∗^*p < 0.05; ^∗∗^*p* < 0.01; ^∗∗∗^*p* < 0.001*.

*STAI-Y1, State Anxiety Inventory-Form Y; STAI-Y2, Trait Anxiety Inventory – Form Y; BDI, Beck Depression Scale; SF-36, Short-Form Health Survey 36; SF-36 PF, physical functioning; SF-36 RP, role limitations due to physical health; SF-36 BP, bodily pain; SF-36 GH, general health; SF-36 EN, energy; SF-36 SF, social functioning; SF-36 RE, role limitations due to emotional problems; SF-36 MH, mental health; SCL-90, Symptom Check List 90; SCL-90 SOM, somatization; SCL-90 O–C, obsessive–compulsive; SCL-90 IS, interpersonal sensitivity; SCL-90 DEP, depression; SCL-90 ANX, anxiety; SCL-90 HOS, hostility; SCL-90 PHOB, phobic anxiety; SCL-90 PAR, paranoid ideation; SCL-90 PSY, psychoticism; PMQ, Personal Meaning Questionnaire; PMQ ED, Eating Disorder personal meaning organization; PMQ DEP, Depressive personal meaning organization; PMQ PHOB, Phobic personal meaning organization; PMQ OBS, Obsessive personal meaning organization*.

Multiple Sclerosis and PH groups are comparable according to age, both within them and when compared to controls. Differently, ALS group significantly differs from the other three groups with regard to age (*p* < 0.01); this data is in accordance with late onset of ALS when compared to MS and PH patients.

When considering educational level, MS and PH patients are comparable among them and with the control sample. ALS patients have a significantly lower level of education with respect to controls (*p* = 0.029), and both MS (*p* = 0.037) and PH (*p* = 0.037) groups.

### Psychological and QoL Features

The comparison between the three neurological populations and the control sample highlights a global condition of psychological stress in the former, with prominent anxiety components depicted. In fact, significant anxiety symptoms where observed in all the three neurological conditions, and more specifically with regard to MS (STAI-Y1: *p* = 0.027, STAI-Y2: *p* = 0.001, SCL-90 ANX: *p* = 0.008), PH (STAI-Y1: *p* = 0.004, STAI-Y2: *p* = 0.003, SCL-90 ANX: *p* = 0.006) and ALS (STAI-Y1: *p* = 0.004, STAI-Y2: *p* = 0.021, SCL-90 ANX: *p* = 0.010) groups.

Thoughts are consistently focused on the clinical condition (SCL-90 PHOB: MS, *p* = 0.037; PH, *p* = 0.036; ALS, *p* < 0.001) and patients seem to present recurring ruminations about health-related issues (SCL-90 O–C: MS, *p* = 0.019; PH, *p* = 0.019; ALS, *p* = 0.019).

Moreover, a significant reduction in social activities is observed in PH and ALS (SF-36 SF: PH, *p* < 0.001; ALS, *p* = 0.008). Higher rates of psychoticism are reported by all neurological samples (SCL-90 PSY: MS, *p* = 0.009; PH, *p* = 0.009; ALS, *p* = 0.009). Somatic expression of the underlying emotional diseases have been reported globally in neurological patients (SCL-90 SOM: MS, *p* = 0.009; PH, *p* = 0.009; ALS, *p* = 0.009).

**FIGURE 1:**
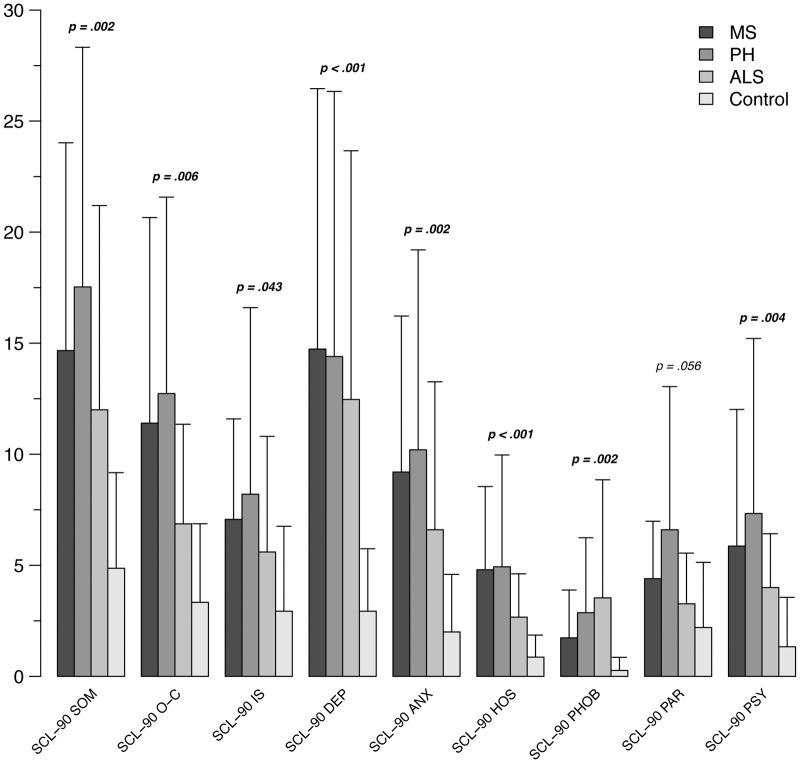
**Mean scores at SCL-90 subscales in the patients groups (ALS, MS, PH) and control subjects**. SCL-90, Symptom Check List 90; SOM, somatization; O–C, obsessive–compulsive; IS, interpersonal sensitivity; DEP, depression; ANX, anxiety; HOS, hostility; PHOB, phobic anxiety; PAR, paranoid ideation; PSY, psychoticism.

Mood of patients, when compared to controls, is globally depressed, with feeling of sadness and substantial pessimism about future (SCL-90 DEP: MS, *p* = 0.004; PH, *p* = 0.005; ALS, *p* < 0.001; BDI: MS, *p* = 0.003; PH, *p* = 0.009; ALS, *p* < 0.001). Moreover, feeling of anger and other-directed hostility are present in all conditions (SCL-90 HOS: MS, *p* = 0.002; PH, *p* = 0.002; ALS, *p* = 0.01) (See **Figure 1**).

**FIGURE 2:**
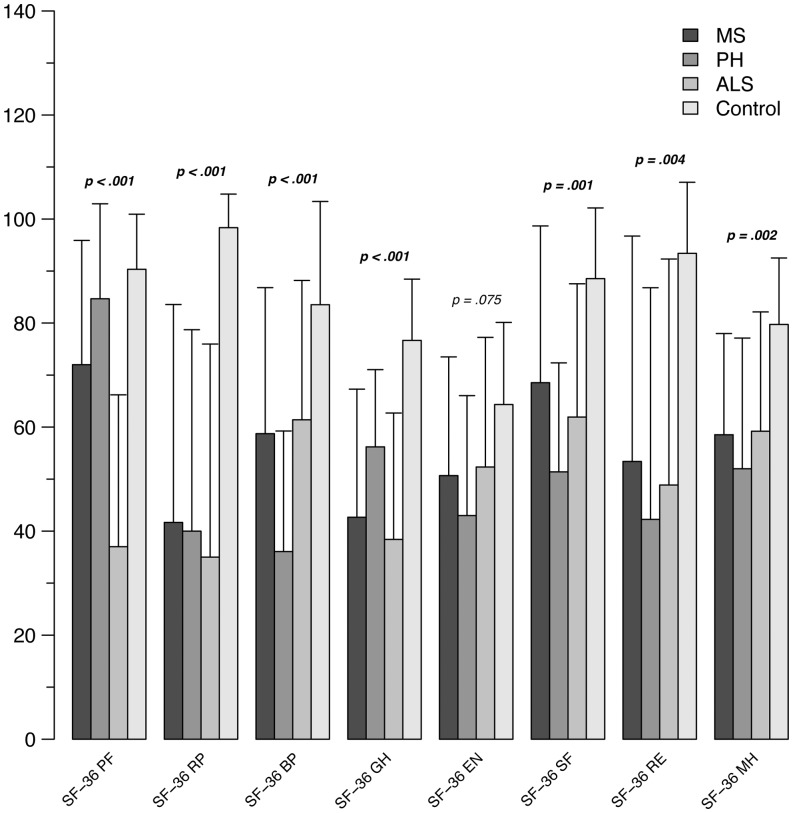
**Mean scores at SF-36 subscales in the patients groups (ALS, MS, PH) and control subjects**. SF-36, Short-Form Health Survey 36; PF, physical functioning; RP, role limitations due to physical health; BP, bodily pain; GH, general health; EN, energy; SF, social functioning; RE, role limitations due to emotional problems; MH, mental health.

As expected, neurological samples show a lower QoL when compared to control group, even if a prevalence of bodily over emotional component has not been recorded (see **Figure 2**). In particular, patients rate their health related condition as less satisfying than controls (SF-36 GH: MS, *p* < 0.001; PH, *p* = 0.013; ALS, *p* < 0.001), with role limitations due to physical and emotional problems in all three conditions (SF-36 RP: MS, *p* = < 0.001; PH, *p* < 0.001; ALS, *p* < 0.001; SF-36 RE: MS, *p* = 0.009; PH, *p* = 0.007; ALS, *p* = 0.009). Moreover, a substantial dissatisfaction concerning MH is highlighted (SF-36 MH: MS, *p* = 0.005; PH, *p* = 0.005; ALS, *p* = 0.017). Limitations in physical activities are reported by all patients (SF-36 PP: MS, *p* = 0.03, PH, *p* < 0.001, ALS, *p* = 0.03). In PH group, a tendency to a reduction in energy and vitality is reported (SF-36 EN: *p* = 0.06).

When comparing the three neurological conditions, different rates of satisfaction with regard to global health-related status are observed, with lower levels reported for ALS with respect to PH (SF-36 GH: PH > ALS, *p* = 0.025). Another relevant result concerns BP, that is rated as more disabling by PH patients (SF-36 BP: PH > ALS: *p* = 0.02; PH > MS: *p* = 0.02; PH vs N, *p* ≤ 0.001), even if also reported by both ALS and MS (SF-36 BP: ALS > N, *p* = 0.03; MS > N, *p* = 0.03).

### Correlations

With regard to controls, correlations concerning relationships between PMQ and other measures showed that in the ED PMO thoughts are characterized by a strong sensitivity to judgment perceived in others (SCL-90 PAR: *r*_s_ = 0.618, *p* = 0.014; SCL-90 O-C: *r*_s_ = 0.765, *p* = 0.001). This aspect is accompanied by a reduction of engagement in social activities and increased feelings of personal inadequacy and inferiority in comparisons with others (SF-36 SF: *r*_s_ = –0.599, *p* = 0.018; SCL-90 IS *r*_s_ = 0.560, *p* = 0.026). Several correlations are also present with distress arising from bodily perceptions (SCL-90 SOM: *r*_s_ = 0.596, *p* = 0.019), increased levels of anxiety or depression (BDI: *r*_s_ = 0.608, *p* = 0.016; SCL-90 DEP: *r*_s_ = 0.678, *p* = 0.006; STAI Y2: *r*_s_ = 0.705, *p* = 0.003; SCL-90 ANX: *r*_s_ = 0.730, *p* = 0.002) and reduction in motor initiative (SF-36 PF: *r*_s_ = –0.801, *p* = 0.000).

In the neurological samples, ED PMO presents with different features with respect to controls. In ALS, higher rates of avoidance behaviors and concerns about their own MH are reported (SCL-90 PHOB: *r*_s_ = 0.571, *p* = 0.026; SF-36 MH: *r*_s_ = –0.517, *p* = 0.049), while in MS a reduction in energy/vitality is revealed (SF-36 EN: *r*_s_ = –0.572, *p* = 0.026). Similarly, in PH a higher destabilization is observed with respect to controls, with higher levels of withdrawal and isolation from others and lower perceived MH with respect to controls (SF-36 MH: *r*_s_ = –0.588, *p* = 0.021; SCL-90 PSY: *r*_s_ = –0.667, *p* = 0.007).

In the control group, the DEP PMO is associated by depressive symptoms with signs of withdrawal from interests, lack of motivation and loss of vital energy, together with isolations (BDI: *r*_s_ = 0.542, *p* = 0.037; SCL-90 DEP: *r*_s_ = 0.571, *p* = 0.026; SCL-90 PSY: *r*_s_ = 0.533, *p* = 0.033). Those findings seem to influence also GH status and engagement in physical activities (SF-36 PF: *r*_s_ = –0.707, *p* = 0.003; SF-36 GH: *r*_s_ = –0.573, *p* = 0.025).

Such aspects are observed also in MS and PH populations; in particular, in the DEP PMO the presence of a neurological disease seems to further reduce interest for environment and social relations (SF-36 SF: MS, *r*_s_ = –0.726, *p* = 0.002; SCL-90 PHOB: *r*_s_ = 0.574, *p* = 0.025; SCL-90 IS: MS and PH, *r*_s_ ≤ 0.767, *p* ≤ 0.007). Thoughts appear characterized by more severe worries (SCL-90 PAR: *r*_s_ ≤ 0.649, *p* ≤ 0.023; SCL-90 O-C: *r*_s_ ≤ 0.648, *p* = 0.016), mainly concerning physical symptoms in MS (SF-36 BP: *r*_s_ = –0.519, *p* = 0.047; SCL-90 SOM: *r*_s_ = 0.566, *p* = 0.028) and mental health in PH (SF-36 MH: *r*_s_ = –0.588, *p* = 0.021). These concerns are accompanied by relevant anxiety in such patients (STAI Y2: *r*_s_ ≤ 0.712, *p* ≤ 0.025; SCL-90 ANX: *r*_s_ ≤ 0.578, *p* ≤ 0.031).

Differently, in ALS subgroup no significant correlations have been observed between PMQ and other questionnaires.

The PHOB PMO in the control group reveals a mindset characterized by distinct concerns for health status and limitations that could arise from emotional disturbances (SF-36 GH: *r*_s_ = –0.589, *p* = 0.021; SF-36 RE: *r*_s_ = –0.561, *p* = 0.029). Patients with such PMO presenting with ALS manifest increased generalized anxiety symptoms (STAI Y1: *r*_s_ = 0.537, *p* = 0.039), while PH patients reveal withdrawal and isolation (SCL-90 PSY: *r*_s_ = 0.689, *p* = 0.005). Both these neurological conditions, share the presence of more frequent concerns that may turn into ruminations (SCL-90 O-C: *r*_s_ ≤ 0.541, *p* ≤ 0.043). Conversely, no correlations have been observed between psychological symptoms in MS and PHOB organization of personality.

No correlations between physical and psychological symptoms and OBS PMO were detected in all groups considered.

### Personal Meaning Questionnaire

Personal Meaning Questionnaire data analysis in MS patients showed higher values of ED (*p* = 0.025) and DEP organizations (*p* = 0.034) when compared to control sample. Similarly, in PH subgroup higher values of ED (*p* = 0.025) and DEP (*p* = 0.034) organizations were observed. Finally, ALS subgroup showed higher rates of PHOB (*p* = 0.050) with respect to controls.

**FIGURE 3:**
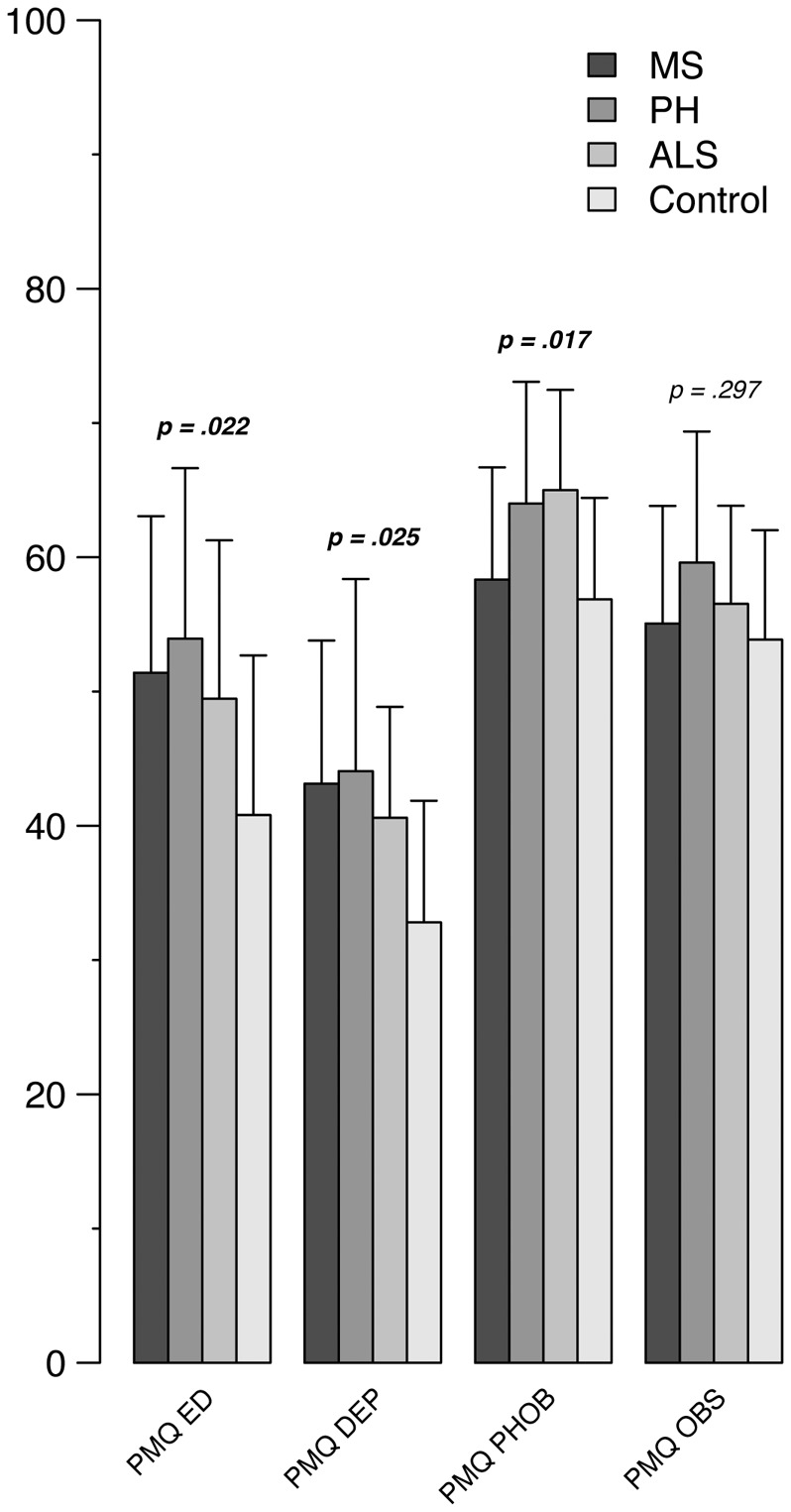
**Distribution of PMO in the three neurological conditions and in control subjects**. PMQ, Personal Meaning Questionnaire; ED, Eating Disorder personal meaning organization; DEP, Depressive personal meaning organization; PHOB, Phobic personal meaning organization; OBS, Obsessive personal meaning organization.

Across all the four conditions, a higher prevalence of PHOB organization has been detected, without significant differences between groups (MS: 66.67%, PH: 80%, ALS: 93.33%, controls: 60%; *p* = 0.14) (see **Figure 3**).

## Discussion

For a long time, personality of neurological patients has been poorly investigated, as well as psychotherapeutic approaches tailored on these populations.

The present study has been aimed to empirically investigate the construct of personal meaning organization developed by Guidano (Guidano, 1987), by employing the PMQ for evaluating three different groups of patients with neurological disease (ALS, MS, PH). In particular, the study aimed to explore, with a specific tool designed for detecting PMO styles, the possible presence of peculiar personality features characterizing the neurological conditions examined.

The main finding concerns the detection, across all clinical conditions, of a higher prevalence of PHOB personality style, with ALS showing a relevant prevalence of such PMO with respect to all other neurological conditions and controls.

However, with respect to controls, in all clinical conditions, PMQ highlighted a tendency to codify experience by means of specific cognitive and emotional patterns.

In fact, in ALS patients, higher prevalence of PHOB personality style, even if not statistically significant, suggests the presence of alexitimic traits typical of phobic organization, consisting of a difficulty in identify emotional experiences and to relate emotional states to somatic perturbations. Such findings are supported by recent literature highlighting disorders of emotional processing in ALS patients (Sedda, 2014). Moreover, these alterations are further supported by neuroimaging and physiological data (Lulé et al., 2005, 2007; Crespi et al., 2014). Probably, a change in basic emotional experience in ALS, with an altered pattern of physiological and behavioral activation toward positive and negative stimuli, could explain the emotional adjustment observed in ALS patients, possibly representing a protective factor against the psychological impact of disability progression.

Nevertheless, such observations could be further interpreted according to cognitive and behavioral changes largely identified in ALS patients (Goldstein and Abrahams, 2013), and to a possible decreased awareness of such changes (lack of insight); the latter should be considered as a further, even if less investigated, feature of the disease (Woolley et al., 2010; Flaherty-Craig et al., 2011).

The significant compliance to medical treatment and personality features often observed in ALS patients could likely arise from the above depicted aspects. Our findings, identifying a higher prevalence of PHOB personality style in ALS, are consistent with Brown and Mueller description (Brown and Mueller, 1970) about patients’ reaction to disease, reporting an autonomous, hard-working behavioral style of active mastery, the use of denial coping, and a tendency to use emotional control to exclude the experience of negative affect from awareness.

In both PH and MS patients, even if the PMO profile more present is represented by PHOB personality style, when compared to controls they show significantly higher values for ED and DEP personality features. Despite such findings, ED and DEP personality traits do not fulfill the completeness necessary to identify a different PMO for such neurological conditions.

Concerning MS population, it’s worth to underline that previous studies mainly focused on the association between specific personality features and presence/severity of psychological and somatic disorders, instead of investigating specific personality profiles characterizing such population; moreover, heterogeneous instruments have been used for such purpose. Finally, a possible confounding effect of the emotional adjustment to the disease when measuring psychological aspects of patients should be taken into account. Thus, further investigation of personality aspects in MS population is needed in order to collect more informative data.

Concerning PH patients, higher values observed in ED traits are in line with previous description of psychological and personality aspects highlighting the presence of an analytic cognitive style in such population (Buonfiglio and Di Sabato, 2011), as could be expected in those persons that tend to be more consciously aware of the evaluative processes generating an emotion, instead of more ‘basic emotions’ (Arciero et al., 2004).

To summarize, our data concerning the above-mentioned conditions do not clearly support disease specific personality styles both for MS and PH, even if the presence of higher values in ED and DEP PMOs suggests the need for further investigations.

The higher prevalence of PHOB personality in the overall sample is in accordance with Guidano’s considerations (Guidano, 1987) about distribution of PMO, showing PHOB one as the most common.

Correlation analysis concerning relationships between PMQ and other questionnaires in the control sample partly support Guidano’s PMO (Guidano, 1987) description and characterization, especially when ED, DEP and PHOB PMOs are considered. The lack of correlational data between Guidano’s OBS PMO and the assessment tools administered arise concerning about such PMO characterization when detected with PMQ.

The above-mentioned considerations can be extended to correlational data concerning PMQ results and the other administered questionnaires in neurological conditions examined, with exception of DEP PMO in ALS and PHOB PMO in MS. As in controls, no significant correlations were detected for OBS PMO in all clinical groups considered.

An interesting finding refers to the construct of Guidano’s PMOs. Accordingly, to what is recorded in clinical practice, where a continuum between normality and pathology is usually observed, the distribution of personality styles across the neurological conditions involved seem to be only exacerbated, and not modified by the occurrence of the disease. Therefore, neurological disease seems to modify the consistency of the observed profile, instead of modifying the prevalence of different PMOs across diseases.

Even if presented results highlight interesting data, they cannot be considered conclusive nor exhaustive with regard to personality stiles in the three neurological groups considered. A deeper analysis of PMQ usability should include larger samples of neurological patients, as well as the comparison with other personality assessment tools and some methodological adjustments aimed to improve PMQ validity and sensitivity. Furthermore, assessing personality profile by means of PMQ, in comparison to other available personality inventories, allows to deeply focus on the complexity of the human experience and not only on specific personality dimensions. Such approach, in our opinion, could possibly better describe the subjective experience and consequences of living with a neurological disease, thus integrating research and clinical perspectives. In fact, despite the common issue of living with a chronic disabling neurological disorder, specific differences emerged in our neurological samples not only with respect to personality profile, but also in relation to psychological symptoms and quality of life aspects (i.e., PH patients suffering more than MS and ALS patients on a variet of psychological measurements).

## Conclusion

Even if the construct of PMO is useful for clinical psychotherapy, the assessment of cognitive-emotional organizations in neurological patients within the frame of cognitive-constructivist frame needs further experimental investigations.

In spite of these limitations, the findings reported in the present study represent the first contribution towards understanding the personality profiles of patients with neurological conditions according to the cognitive-constructivist theory. We suggest that adequately assessing these profiles in clinical practice may be relevant to plan pharmacological and/or psychological treatment tailored on each patient’s characteristics.

## Author Contributions

BP was responsible for study design and in writing the manuscript; LC contribute in writing the manuscript; AL was responsible for ALS patients assessment; FS was responsible for healthy controls assessment; AF was responsible for statistical analysis; AC was responsible for patients’ neurological examination; MG was responsible for PH patients assessment; VR was responsible for MS patients assessment; RP contribute in designing the study; RA and VS critically revised the manuscript. All the authors approved the final manuscript.

## Conflict of Interest Statement

The authors declare that the research was conducted in the absence of any commercial or financial relationships that could be construed as a potential conflict of interest. The reviewer SZ declared their share affiliation and past co-authorship with several of the authors BP, LC, AL, FS, AF, AC, and VS to the handling Editor, who ensured that the process met the standards of a fair and objective review.

## References

[B1] Aaseth K. Grande R. B. Leikness K. A. Benth J. Š. Lundtqvist C. Russell M. B. (2011). Personality traits and psychological distress in persons with chronic tension-type headache. The Akershus study of chronic headache. *Acta Neurol. Scand.* 124 375–382. 10.1111/j.1600-0404.2011.01490.x 22017633

[B2] Abbate-Daga G. Fassino S. Lo Giudice R. Rainero I. Gramaglia C. Marech L. (2007). Anger, depression and personality dimensions in patients with migraine without aura. *Psychother. Psychosom.* 76 122–128. 10.1159/000097971 17230053

[B3] Aben I. Denollet J. Lousberg R. Verhey F. Wojciechowski F. Honig A. (2002). Personality and vulnerability to depression in stroke patients: a 1-year prospective follow-up study. *Stroke* 33 2391–2395. 10.1161/01.STR.0000029826.41672.2E 12364726

[B4] Apolone G. Mosconi P. Ware J. E. Jr. (1997). *Questionario sullo Stato di Salute SF-36. Manuale d’uso e Guida all’interpretazione dei Risultati*. Milano: Guerini e Associati.

[B5] Arciero G. (2002). *Studi e Dialoghi sull’identità Personale*. Turin: Bollati Boringhieri.

[B6] Arciero G. Gaetano P. Maselli P. Gentili N. (2004). “Identity, personality and emotional regulation,” in *Cognition & Psychotherapy*, 2nd Edn, eds Freeman A. Mahoney M. J. Devito P. Martin D. (New York, NY: Springer Publishing Company).

[B7] Baretz R. M. Stephenson G. R. (1981). Emotional responses to multiple sclerosis. *Psychosomatics* 22 117–127. 10.1016/S0033-3182(81)73546-1 7208778

[B8] Beck A. T. Rush A. J. Shaw B. F. Emery G. (1979). *Cognitive Therapy of Depression.* New York, NY: Guilford Press.

[B9] Beeldman E. Raaphorst J. Klein Twennaar M. de Visser M. Schmand B. A. de Haan R. J. (2015). The cognitive profile of ALS: a systematic review and meta-analysis update. *J. Neurol. Neurosurg. Psychiatry* 87 611–619. 10.1136/jnnp-2015-310734 26283685

[B10] Benedict R. H. Schwartz C. E. Duberstein P. Healy B. Hoogs M. Bergsland N. (2013). Influence of personality on the relationship between gray matter volume and neuropsychiatric symptoms in multiple sclerosis. *Psychosom. Med.* 75 253–261. 10.1097/PSY.0b013e31828837cc 23504242

[B11] Berry J. K. Drummond P. D. (2014). Does attachment anxiety increase vulnerability to headache? *J. Psychosom. Res.* 76 113–120. 10.1016/j.jpsychores.2013.11.018 24439686

[B12] Bertolino A. Arciero G. Rubino V. Latorre V. De Candia M. Mazzola V. (2005). Variation of human amygdala response during threatening stimuli as a function of 5’HTTLPR genotype and personality style. *Biol. Psychiatry* 57 1517–1525. 10.1016/j.biopsych.2005.02.031 15953488

[B13] Blomkvist V. Hannerz J. Katz L. Theorell T. (2002). Coping style and social support in men and women suffering from cluster headache or migraine. *Headache* 42 178–184. 10.1046/j.1526-4610.2002.02049.x 11903540

[B14] Bottiroli S. Viana M. Sances G. Ghiotto N. Guaschino E. Galli F. (2016). Psychological factors associated with failure of detoxification treatment in chronic headache associated with medication overuse. *Cephalalgia* 36 1356–1365. 10.1177/0333102416631960 26879321

[B15] Bragazzi N. L. (2013). The gap in the current research on the link between health locus of control and multiple sclerosis: lessons and insights from a systematic review. *Mult. Scler. Int.* 2013:972471 10.1155/2013/972471 PMC3586487 23476777

[B16] Brooks B. R. Miller R. G. Swash M. Munsat T. L. World Federation of Neurology Research Group on Motor Neuron Disease (2000). El Escorial revisited: revised criteria for the diagnosis of amyotrophic lateral sclerosis. *Amyotroph. Lateral Scler. Other Motor Neuron Disord.* 1 293–299. 10.1080/146608200300079536 11464847

[B17] Brown W. A. Mueller P. S. (1970). Psychological function in individuals with amyotrophic lateral sclerosis (ALS). *Psychosom. Med.* 32 141–152. 10.1097/00006842-197003000-00002 4392415

[B18] Buonfiglio M. Di Sabato F. (2011). Analytic cognitive style in cluster headache. *Neurol. Sci.* 32 875–881. 10.1007/s10072-011-0730-1 21850427

[B19] Caprara G. V. Barbaranelli C. Borgogi L. Perugini M. (1993). The big five questionnaire : a new questionnaire for the measurement of the five factor model. *Pers. Individ. Dif.* 15 281–288. 10.3389/fpsyg.2014.01005

[B20] Cloninger C. R. Przybeck T. R. Svrakic D. M. Wetzel R. D. (1994). *The Temperament and Character Inventory (TCI): A guide to its development and use*. St. Louis, MO: Center for Psychobiology and Personality, Washington University.

[B21] Crespi C. Cerami C. Dodich A. Canessa N. Arpone M. Iannaccone S. (2014). Microstructural white matter correlates of emotion recognition impairment in Amyotrophic Lateral Sclerosis. *Cortex* 53 1–8. 10.1016/j.cortex.2014.01.002 24534360

[B22] Derogatis L. R. Lipman R. S. Covi L. (1973). SCL-90: an outpatient psychiatric rating scale – preliminary report. *Psychopharmacol. Bull.* 9 13–28. 4682398

[B23] Even C. Friedman S. Dardennes R. Zuber M. Guelfi J. D. (2004). Prevalence of depression in multiple sclerosis: a review and meta-analysis. *Rev. Neurol.* 160 917–925. 10.1016/S0035-3787(04)71073-5 15492718

[B24] Fazekas C. Khalil M. Enzinger C. Matzer F. Fuchs S. Fazekas F. (2013). No impact of adult attachment and temperament on clinical variability in patients with clinically isolated syndrome and early multiple sclerosis. *Clin. Neurol. Neurosurg.* 115 293–297. 10.1016/j.clineuro.2012.05.022 22721773

[B25] Feinstein A. (2004). The neuropsychiatry of multiple sclerosis. *Can. J. Psychiatry* 49 157–163. 10.1177/070674370404900302 15101497

[B26] Feinstein A. (2011). Multiple sclerosis and depression. *Mult. Scler. J.* 17 1276–1281. 10.1177/1352458511417835 22058085

[B27] Fishman I. Benedict R. H. Bakshi R. Priore R. Weistock-Guttman B. (2004). Construct validity and frequency of euphoria sclerotica in multiple sclerosis. *J. Neuropsychiatry Clin. Neurosci.* 16 350–356. 10.1176/jnp.16.3.350 15377743

[B28] Flaherty-Craig C. V. Brothers A. Yang C. Svoboda R. Simmons Z. (2011). Declines in problem solving and anosognosia in amyotrophic lateral sclerosis: application of Guilford’s structure of intellect theory. *Cogn. Behav. Neurol.* 24 26–34. 10.1097/WNN.0b013e3182138454 21467921

[B29] Goldstein L. H. Abrahams S. (2013). Changes in cognition and behaviour in amyotrophic lateral sclerosis: nature of impairment and implications for assessment. *Lancet Neurol.* 12 368–380. 10.1016/S1474-4422(13)70026-7 23518330

[B30] Grossman A. B. Levin B. E. Bradley W. G. (2006). Premorbid personality characteristics of patients with ALS. *Amyotroph. Lateral Scler.* 7 27–31. 10.1080/14660820510012004 16546756

[B31] Guidano V. F. (1981). *The Self in Process.* New York, NY: Guilford.

[B32] Guidano V. F. (1987). *Complexity of the Self.* New York, NY: Guilford.

[B33] Guidano V. F. Liotti G. (1983). *Cognitive Processes and Emotional Disorder.* New York, NY: Guilford Press.

[B34] Hathaway S. R. McKinley J. C. (1997). *MMPI-2 – Minnesota Multiphasic Personality Inventory–2.* Firenze: Organizzazioni Speciali.

[B35] Headache Classification Committee of the International Headache Society (1988). Classification and diagnostic criteria for headache disorders, cranial neuralgias and facial pain. *Cephalalgia* 8 1–96. 3048700

[B36] Headache Classification Committee of the International Headache Society (2004). The international classification of headache disorders: 2nd edition. *Cephalalgia* 24(Suppl. 1), 9–160. 14979299 10.1111/j.1468-2982.2003.00824.x

[B37] Houpt J. L. Gould B. S. Norris F. H. Jr. (1977). Psychological characteristics of patients with amyotrophic lateral sclerosis. *Psychosom. Med.* 39 299–303. 10.1097/00006842-197709000-00003 910010

[B38] Incerti C. C. Argento O. Pisani V. Magistrale G. Sabatello U. Caltagirone C. (2016). A more in-depth interpretation of MMPI-2 in MS patients by using Harris and Lingoes subscales. *Appl. Neuropsychol. Adult* 10.1080/23279095.2016.1197128 [Epub ahead of print]. 27355486

[B39] Incerti C. C. Magistrale G. Argento O. Pisani V. Di Battista G. Ferraro E. (2015). Occupational stress and personality traits in multiple sclerosis: a preliminary study. *Mult. Scler. Relat. Disord.* 4 315–319. 10.1016/j.msard.2015.06.001 26195049

[B40] Invernizzi G. Landoni M. G. Oggioni C. R. G. Bellini M. Perucchetti G. Lamura A. (1988). “Aspetti psicopatologici nella sclerosi multipla,” in *Proceedings of the International Multiple Sclerosis Conference, Roma: An Update Multiple Sclerosis* Vol. 1989 (Bologna: Monduzzi), 517–520.

[B41] Johansson S. Gottberg K. Kierkegaard M. Ytterberg C. (2016). Variations in and predictors of the occurrence of depressive symptoms and mood symptoms in multiple sclerosis: a longitudinal two-year study. *BMC Neurol.* 16:32 10.1186/s12883-016-0551-1 PMC4779263 26944059

[B42] Korostil M. Feinstein A. (2007). Anxiety disorders and their clinical correlates in multiple sclerosis patients. *Mult. Scler.* 13 67–72. 10.1177/1352458506071161 17294613

[B43] Krampe H. Bartels C. Victorson D. Enders C. K. Beaumont J. Cella D. (2008). The influence of personality factors on disease progression and health-related quality of life in people with ALS. *Amyotroph. Lateral Scler.* 9 99–107. 10.1080/17482960701875805 18428002

[B44] Kurt A. Nijboer F. Matuz T. Kübler A. (2007). Depression and anxiety in individuals with amyotrophic lateral sclerosis: epidemiology and management. *CNS Drugs* 21 279–291. 10.2165/00023210-200721040-00003 17381183

[B45] Lublin F. D. Reingold S. C. (1996). Defining the clinical course of multiple sclerosis: results of an international survey. *Neurology* 46 907–911. 10.1212/WNL.46.4.907 8780061

[B46] Lublin F. D. Reingold S. C. Cohen J. A. Cutter G. R. Sørensen P. S. Thompson A. J. (2014). Defining the clinical course of multiple sclerosis. The 2013 revisions. *Neurology* 83 278–286. 10.1212/WNL.0000000000000560 24871874 PMC4117366

[B47] Lulé D. Diekmann V. Anders S. Kassubek J. Kübler A. Ludolph A. C. (2007). Brain responses to emotional stimuli in patients with amyotrophic lateral sclerosis (ALS). *J. Neurol.* 254 519–527. 10.1007/s00415-006-0409-3 17401515

[B48] Lulé D. Häcker S. Ludolph A. Birbaumer N. Kübler A. (2008). Depression and quality of life in patients with amyotrophic lateral sclerosis. *Dtsch. Arztebl. Int.* 105 397–403. 10.3238/arztebl.2008.0397 19626161 PMC2696844

[B49] Lulé D. Kurt A. Jürgens R. Kassubek J. Diekmann V. Kraft E. (2005). Emotional responding in amyotrophic lateral sclerosis. *J. Neurol.* 252 1517–1524. 10.1007/s00415-005-0907-8 15977000

[B50] Lulé D. Pauli S. Altintas E. Singer U. Merk T. Uttner I. (2012). Emotional adjustment in amyotrophic lateral sclerosis (ALS). *J. Neurol.* 259 334–341. 10.1007/s00415-011-6191-x 21808983

[B51] Manack A. N. Buse D. C. Lipton R. B. (2011). Chronic migraine: epidemiology and disease burden. *Curr. Pain Headache Rep.* 15 70–78. 10.1007/s11916-010-0157-z 21063918

[B52] McDonald W. I. Compston A. Edan G. Goodkin D. Hartung H. P. Lublin F. D. (2001). Recommended diagnostic criteria for multiple sclerosis: guidelines from the International Panel on the diagnosis of multiple sclerosis. *Ann. Neurol.* 50 121–127. 10.1002/ana.1032 11456302

[B53] Merkelbach S. König J. Sittinger H. (2003). Personality traits in multiple sclerosis (MS) patients with and without fatigue experience. *Acta Neurol. Scand.* 17 195–201. 10.1034/j.1600-0404.2003.02037.x 12614312

[B54] Minden S. L. Orav J. Reich P. (1987). Depression in multiple sclerosis. *Gen. Hosp. Psychiatry* 9 426–434. 10.1016/0163-8343(87)90052-1 3692149

[B55] Mohamadi A. Davoodi-Makinejada M. Azimib A. Nafissic S. (2016). Personality characteristics in MS patients: the role of avoidant personality. *Clin. Neurol. Neurosurg.* 144 23–27. 10.1016/j.clineuro.2016.02.035 26963086

[B56] Mohr D. C. Epstein L. Luks T. L. Goodkin D. Cox D. Goldberg A. (2003). Brain lesion volume and neuropsychological function predict efficacy of treatment for depression in multiple sclerosis. *J. Consult. Clin. Psychol.* 71 1017–1024. 10.1037/0022-006X.71.6.1017 14622077

[B57] Mongini F. Ibertis F. Barbalonga E. Raviola F. (2000). MMPI–2 profiles in chronic daily headache and their relationship to anxiety levels and accompanying symptoms. *Headache* 40 466–472. 10.1046/j.1526-4610.2000.00070.x 10849043

[B58] Muñoz I. Hernández M. S. Santos S. Jurado C. Ruiz L. Toribio E. (2016). Personality traits in patients with cluster headache: a comparison with migraine patients. *J Headache Pain* 17:25 10.1186/s10194-016-0618-9 PMC4791411 26975362

[B59] Nelson L. D. Elder J. T. Tehrani P. Groot M. Jantje L. (2003). Measuring personality and emotional functioning in multiple sclerosis: a cautionary note. *Arch. Clin. Neuropsychol.* 18 419–429. 10.1016/S0887-6177(02)00158-0 14591455

[B60] Norris L. Que G. Bayat E. (2010). Psychiatric aspects of amyotrophic lateral sclerosis (ALS). *Curr. Psychiatry Rep.* 12 239–245. 10.1007/s11920-010-0118-6 20425287

[B61] Norton P. J. Asmundson G. J. (2004). Anxiety sensitivity, fear, and avoidance behaviour in headache pain. *Pain* 111 218–223. 10.1016/j.pain.2004.06.018 15327826

[B62] Patten S. B. Beck C. A. Williams J. V. A. Barbui C. Metz L. M. (2003). Major depression in multiple sclerosis: a population-based perspective. *Neurology* 61 1524–1527. 10.1212/01.WNL.0000095964.34294.B4 14663036

[B63] Peters P. K. Swenson W. M. Mulder D. W. (1978). Is there a characteristic personality profile in amyotrophic lateral sclerosis? A minnesota multiphasic personality inventory study. *Arch. Neurol.* 35 321–322. 10.1001/archneur.1978.00500290067011 646685

[B64] Picardi A. (2003). First steps in the assessment of cognitive-emotional organisation within the framework of guidano’s model of the self. *Psychother. Psychosom.* 72 363–365. 10.1159/000073037 14526143

[B65] Picardi A. Gaetano P. Toni A. Caroppo E. (2004). Sostegno alla teoria delle “organizzazioni di significato personale” da altre elaborazioni teoriche nell’area della personalità: uno studio di validità convergente del QSP. *Riv. Psichiatr.* 39 112–124.

[B66] Picardi A. Mannino G. (2001). Le “organizzazioni di significato personale”: verso una validazione empirica. *Riv. Psichiatr.* 36 224–233.

[B67] Picardi A. Mannino G. Arciero G. Gaetano P. Pilleri M. F. Arduini L. (2003). Costruzione e validazione del QSP, uno strumento per la valutazione dello stile di personalità secondo la teoria delle “organizzazioni di significato personale”. *Riv. Psichiatr.* 38 13–34.

[B68] Polman C. H. Reingold S. C. Banwell B. Clanet M. Cohen J. A. Filippi M. (2011). Diagnostic criteria for multiple sclerosis: 2010 revisions to the Mc Donald criteria. *Ann. Neurol.* 69 292–302. 10.1002/ana.22366 21387374 PMC3084507

[B69] R Core Team (2015). *R: A Language and Environment for Statistical Computing*. Vienna: R Foundation for Statistical Computing Available at: http://www.R-project.org/

[B70] Rabins P. V. Brooks B. R. O’Donnel P. Pearlson G. D. Moberg P. Jubelt B. (1986). Structural brain correlates of emotional disorder in multiple sclerosis. *Brain* 109 585–597. 10.1093/brain/109.4.585 3730806

[B71] Rubino V. Blasi G. Latorre V. Fazio L. d’Errico I. Mazzola V. (2007). Activity in medial prefrontal cortex during cognitive evaluation of threatening stimuli as a function of personality style. *Brain Res. Bull.* 74 250–257. 10.1016/j.brainresbull.2007.06.019 17720547

[B72] Sadovnick A. D. Eisen K. Ebers G. C. Paty D. W. (1991). Cause of death in patient attending multiple sclerosis clinics. *Neurology* 41 1193–1196. 10.1212/WNL.41.8.1193 1866003

[B73] Schreiber H. Lang M. Kiltz K. Lang C. (2015). Is personality profile a relevant determinant of fatigue in multiple sclerosis? *Front. Neurol.* 6:2 10.3389/fneur.2015.00002 PMC4316719 25699007

[B74] Sedda A. (2014). Disorders of emotional processing in amyotrophic lateral sclerosis. *Curr. Opin. Neurol.* 27 659–665. 10.1097/WCO.0000000000000147 25333604

[B75] Silani V. Messina S. Poletti B. Morelli C. Doretti A. Ticozzi N. (2011). The diagnosis of Amyotrophic lateral sclerosis in 2010. *Arch. Ital. Biol.* 149 5–27. 21412713 10.4449/aib.v149i1.1260

[B76] Spielberger C. D. Gorsuch R. L. Lushene R. E. (1989). *STAI. State-Trait Anxiety Inventory – Forma Y.* Firenze: Giunti OS Organizzazioni speciali.

[B77] Strober L. B. (2016). Personality in multiple sclerosis (MS): impact on health, psychological well-being, coping, and overall quality of life. *Psychol. Health Med.* 18 1–10. 10.1080/13548506.2016.1164321 PMC5214537 26987417

[B78] Torelli P. Abrignani G. Castellini P. Lambru G. Manzoni G. M. (2008). Human psyche and headache: tension-type headache. *Neurol. Sci.* 29 S93–S95. 10.1007/s10072-008-0896-3 18545906

[B79] Trojano M. Paolicelli D. (2001). The differential diagnosis of multiple sclerosis: classification and clinical features of relapsing and progressive neurological syndromes. *Neurol. Sci.* 22 S98–S102. 10.1007/s100720100044 11794488

[B80] Verdolini N. De Giorgio G. Moretti P. Piselli M. Quartesan R. (2015). The psychosomatic spectrum: a clinical-analytic survey of the relationship between eating disorders and migraine. *Psychiatr. Danub.* 27(Suppl. 1), S332–S335. 26417790

[B81] Waldron E. J. Barrash J. Swenson A. Tranel D. (2014). Personality disturbances in amyotrophic lateral sclerosis: a case study demonstrating changes in personality without cognitive deficits. *J. Int. Neuropsychol. Soc.* 20 764–771. 10.1017/S1355617714000459 24854881 PMC4429900

[B82] Withlock F. A. Siskind M. M. (1980). Depression as a major symptom of multiple sclerosis. *J. Neurol. Neurosurg. Psychiatry* 43 861–865. 10.1136/jnnp.43.10.861 7441263 PMC490704

[B83] Woolley S. C. Moore D. H. Katz J. S. (2010). Insight in ALS: awareness of behavioral change in patients with and without FTD. *Amyotroph. Lateral Scler.* 11 52–56. 10.3109/17482960903171110 19714539

[B84] Yucel B. Kora K. Ozyalçìn S. Alçalar N. Ozdemir O. Yucel A. (2002). Depression, automatic thoughts, alexithymia, and assertiveness in patients with tension-type headache. *Headache* 42 194–199. 10.1046/j.1526-4610.2002.02051.x 11903542

[B85] Zarbo I. R. Minacapelli E. Falautano M. Demontis S. Carpentras G. Pogliatti M. (2016). Personality traits predict perceived health-related quality of life in persons with multiple sclerosis. *Mult. Scler.* 22 551–558. 10.1177/1352458515594045 26163067

